# Evaluation of Sleep Behavior and the Use of Sleep Aids among Adults Living in Saudi Arabia: A Cross-Sectional Study

**DOI:** 10.3390/clockssleep5030035

**Published:** 2023-09-11

**Authors:** Ahmed Aldhafiri, Nawaf Almutairi, Mohammed Alharbi, Abdullah Aloufi, Abdulaziz Hakeem, Abdulmalik Kattan, Fahad Alzahrani

**Affiliations:** 1 Pharmacology and Toxicology Department, College of Pharmacy, Taibah University, Al-Madinah al-Munawara 42353, Saudi Arabia; 2 College of Pharmacy, Taibah University, Al-Madinah al-Munawara 42353, Saudi Arabia; 3 Clinical and Hospital Pharmacy Department, College of Pharmacy, Taibah University, Al-Madinah al-Munawara 42353, Saudi Arabia

**Keywords:** sleep behaviors, sleep aids, sleep disorders

## Abstract

A negative attitude toward sleep has greatly affected sleep habits. In addition to contributing to physical and metabolic disorders, poor sleep quality may cause emotional disturbances. This study aimed to measure sleep behavior and factors contributing to poor sleep quality in the Madinah region, Saudi Arabia. We also assessed whether the use of sleeping aids improved peoples sleep. Three hundred and ninety-nine adults in the Madinah region of Saudi Arabia participated in this cross-sectional study. Three data domains were collected using an online questionnaire between 30 January and 26 April 2022. In the first domain, the characteristics of participants were discussed. In the second domain, questions about sleep behavior were asked. In the third domain, we examined the types, frequency, and impact of sleep aid use. Out of the 399 participants, 154 (38.59%) reported sleep problems. A total of 64.94% of the 154 participants blamed stress as the leading cause of their sleep disorders, and 74.68% of those with sleep problems reported reduced productivity. Among those who reported having sleep problems, 46.10% used sleep aids, with Panadol night (antihistamine) being the most used, 49.30%, followed by Melatonin at 39.44%. Sleep quality improved by 67.6% among those who used sleep aids. A total of 71.8% of the participants think it is not safe to use sleep aids in the long term. Our findings suggest that sleep problems are a prevalent concern in Madinah, Saudi Arabia, and even though the use of sleep aids improved sleep quality, it should be considered an emerging and important public health objective in Saudi Arabia. Further studies are needed to evaluate sleep quality and the level of sleep aid usage among other Saudi Arabian regions.

## 1. Introduction

Sleep is an essential and repeated physiological process that regenerates physical and psychological well-being [1]. An inadequate amount of sleep or poor sleep quality may lead to several physical, psychological, and metabolic disorders such as diabetes, increased risk of heart disease, depression, and increased risk of suicide [2,3,4]. Many categories fall within the term sleep disorder, including insomnia, which is the unwanted experience of difficulty sleeping, which can be acute, intermittent, or chronic. Also, sleep apnea is characterized by repeated episodes of upper airway closure during sleep that results in recurrent sleep awakening [5,6]. A previous survey indicated that sleep patterns differ depending on the region and the culture [7]. The consequences of poor sleep quality not only affect physical and psychological health. Furthermore, it can also lead to defective productivity and social activity [8].

Some epidemiological and experimental studies carry evidence that exercise has a sleep-promoting effect. Additionally, some herbs, such as chamomile, appear to be a relatively safe option for improving sleep quality in the short term [9,10]. Pharmacological interventions also play an essential role in the management of sleep disorders. The most commonly used interventions include antihistamines such as diphenhydramine and chlorpheniramine, some complements such as Melatonin and 5-Hydroxytryptophan, and some prescription drugs including benzodiazepines [11,12,13,14].

Globally, the prevalence rate of sleep disorders is 27.3%, while in Saudi Arabia, one study suggested that 61.6% of the Saudi population have or may have sleep disorders [15,16]. Because we have a higher prevalence of sleep disorders than the worldwide population, this study measured sleeping behaviors and the factors that may contribute to poor sleep quality among adults living in the Madinah region, Saudi Arabia. Moreover, we assessed the use of sleeping aids and whether it improved peoples sleep quality.

## 2. Results

### 2.1. Characteristics of the Participants (N = 399)

Table 1 represents the characteristics of the participants. Out of the 403 sent questionnaires, 399 agreed to participate in the study. A total of 31.33% of them are males, while 68.67% are females. A total of 66.92% of the participants had little or no exercise, 19.55% did regular daily activity, 10.53% did regular-to-moderate exercise, and only 3.01% did intense exercise. A total of 22.81% of the participants had a high school degree or lower, 9.52% had a diploma degree, 60.65% had a bachelor’s degree, 6.52% had a master’s degree, and only 0.50% had a Ph.D. degree. A total of 24.06% were students, and 39.10% were working while 36.84% were not. A total of 27.07% spent less than 4 h studying or working, 43.11% spent between 4 and 7 h studying or working, 25.56% spent 7 to 10 h studying or working, and only 4.26% spent more than 10 h studying or working.

Most of the participants (94.24%) were Saudis, while 5.76% of them were non-Saudis. A total of 30.58% of the participants were single, most were married (62.41%), 6.27% were divorced, and only 0.75% were widowed. A total of 4.01% of the participants slept less than 4 h, 67.92% slept 4–7 h, 26.57% slept 7–10 h on a typical day, and only 1.50% slept more than 10 h. A total of 24.06% of the participants usually went to sleep during the day, while 75.94% usually went to sleep at night.

A total of 59.90% of the participants suffered from continuous sleeping awakening, while the rest of the participants did not suffer. A total of 27.57% of the participants took less than 30 min to fall asleep, 47.37% took 30 to 60 min, and 25.06% took more than 60 min. A total of 42.36% of the participants did not take a nap during the day, 17.54% of them had intermittent napping during (week/month) when needed, 10.03% of them took a nap for less than 30 min, 16.29% of them took a nap for 30 min to 1.5 h, 11.53% of them had a nap for 2–3 h during the day, and only 2.26% of them took a nap for more than 3 h. A total of 38.59% of the participants had trouble sleeping, while the rest had no problem.

### 2.2. Characteristics of Sleeping Problems and Using a Sleeping Aid

Table 2 showed that one hundred and fifty-four of the participants had sleeping trouble. The most common cause of sleeping trouble was due to stress (64.94%), followed by wasting time on their phone prior to sleep (37.01%), coffee and tea drinking (33.12%), late activities (19.48%), work and studying (14.94%), followed by health conditions (11.04%), and 7.79% were due to other causes. The productivity of the participants was affected by 74.68% of those who had sleeping trouble, while 25.32% were not affected. A total of 85.06% never consulted a healthcare professional, while 14.94% did. A total of 46.10% of the participants who had difficulty sleeping used sleep aids, while 53.90% did not.

### 2.3. The Association between Having Sleep Troubles and the Characteristics of Participants

The chi-square and Fisher’s exact tests were used to study the association between having sleeping troubles and the characteristics of the participants in Table 3. Five demographic variables demonstrated a statistically significant association with sleeping troubles. Sleep duration showed a statistically significant difference. A total of 81% of participants with sleep troubles sleep less than 4 h, 41.6% sleep between 4 and 7 h, 25% sleep between 7 and 10 h, and 50% sleep more than 10 h, *p*-value < 0.001. The sleeping time showed a statistically significant difference. A total of 55.3% of participants who go to sleep during the day have sleeping troubles, while only 33.8% of participants who go to sleep at night have sleeping troubles, *p*-value < 0.001. Continues sleep awakening showed a statistically significant difference. A total of 53.8% of participants who suffer from continuous sleep awakening have sleeping troubles, while only 16.6% of participants who do not suffer from continuous sleep have sleeping troubles, *p*-value < 0.001. Taking a nap during the day showed a statistically significant difference; 55.6% of participants who take a nap for more than 3 h have sleeping troubles, 51.1% of participants who take a nap from 2 to 3 h have sleeping troubles, 43.1% of participants who do not take a nap have sleeping troubles, 40% of participants who take a nap less than half an hour have sleeping troubles, 28.6% of participants who take a nap when needed have sleeping troubles, and only 28.1% of participants who take a nap from 30 min to 1.5 h have sleeping troubles, *p*-value = 0.044. A total of 66% of participants who take more than an hour to fall asleep have sleeping troubles, 32.6% of participants who take between 30 and 60 min to fall asleep have sleeping troubles, while 25% of people who take less than 30 min to fall asleep have sleeping troubles, *p*-value < 0.001.

### 2.4. Multiple Logistic Regression for the Factors Associated with Having Sleeping Problems

The association between having sleeping problems and other factors was studied using multiple logistic regression in Table 4. Participants who take a nap when needed are less likely to have sleeping troubles than participants who do not, OR = 0.52, *p*-value = 0.044. Participants who sleep at night are less likely to have sleeping troubles than participants who sleep during the day, OR = 0.41, *p*-value = 0.001.

### 2.5. Characteristics of Using Sleeping Aids

Table 5 represents that the most common sleep aids used were Panadol night (antihistamine) (49.30%), Melatonin (39.44%), herbs and other supplements (23.94%), prescription drugs (11.27%), and 9.86% used other sleep aids. The frequency of using sleep aid was as follows: 56.34% used it as needed, 28.17% used it on a daily basis, 1.41% used it monthly, and 4.23% stopped using it. Sleep aids improved sleep quality in 67.6% of people with sleeping problems, while 32.4% of their sleep quality was not affected. A total of 28.2% of the participants think using a sleep aid for the long term is safe, and 71.8% think it is not. A total of 38% of participants did notice some side effects, while 62% did not. A total of 42.3% of participants recommend using a sleep aid, while 57.7% do not. 

### 2.6. The Association between Using Sleep Aids and Characteristics of Participants in Those Having Sleeping Problems

The chi-square test and Fisher’s exact test were used to study the difference between using a sleeping aid and the characteristics of the participants in Table 6. Work status showed a statistically significant association with the use of sleeping aids among those having sleep problems. While 26.3% of students use sleep aids, the percentage of those using sleep aids is 50.8% among working individuals and 54.9% among non-working individuals, *p*-value = 0.017. The duration to fall asleep showed a statistically significant association with the use of sleeping aids among those having sleep problems. While the percentage of participants using sleep aids is 44.4% among participants who stay awake for less than 30 min to fall asleep, the percentage of participants using sleep aids is 31.1% among participants who stay awake between 30 and 60 min. The percentage of participants using sleep aids is 60.6% among participants who stay awake more for than 60 min before they fall asleep, *p*-value = 0.003. Healthcare professional consultation showed a statistically significant association with the use of sleeping aids among those having sleep problems. While 87.0% of the participants who consulted a health care professional about their sleep problems use sleep aids, the percentage of participants using sleep aids who did not consult a health care professional about their sleep problems is 38.9%, *p*-value < 0.001.

### 2.7. Multiple Logistic Regression for the Factors Associated with Using Sleep Aids among Those Having Sleeping Problems

Table 7 presented the association between using sleep aids among those having sleeping problems and other factors was studied using multiple logistic regression. Work status, time to fall asleep, and consulting a healthcare professional showed statistical significance. Working participants and not-working participants are associated with higher odds of using sleeping aids as compared to students (OR = 2.88, 95% CI for OR: = 1.10, 7.55, *p*-value = 0.032) and (OR = 4.31, 95% CI for OR: = 1.56, 11.91, *p*-value = 0.005), respectively. Taking between 30 and 60 min to fall asleep is associated with lower odds of using sleeping aids as compared to taking more than 60 min to fall asleep (OR = 0.27, 95% CI for OR: = 0.12, 0.61, *p*-value = 0.002). Consulting a health care professional about sleep problems is associated with higher odds of using sleeping aids than not consulting a health care professional (OR = 10.96, 95% CI for OR: = 2.87, 41.87, *p*-value < 0.001).

## 3. Discussion

To our knowledge, this is the first study to assess sleep behavior and sleeping aid usage among adults in the Madinah region of Saudi Arabia. The results indicate a high prevalence of sleep problems, with 38.59% of adults in Madinah reporting such issues.

Based on the results, the most common factors contributing to sleep problems were stress, followed by phone usage, coffee and tea drinking, and late-night activities. The term stress is general and may be related to various conditions such as work, study, financial, and family stress. This may be one of the reasons why it was the most chosen factor. Moreover, regarding sleep problems associated with using the phone before sleep, our results are consistent with the previous studies, which showed that the blue light reflected from the phone affects the production of Melatonin, the responsible hormone for sleeping [17,18]. Regarding coffee and tea drinking, the results are consistent with previous studies, which showed that caffeine frequently affected sleep quality, increased sleep latency, decreased total sleep time, and decreased sleep efficiency [19]. The influence of late-night activity on sleep quality largely depends on culture and lifestyle. In Saudi Arabia, many lifestyle practices are performed at night or until midnight, such as weddings and family and friends’ gatherings, which may contribute to poor sleep quality. A total of 74.68% of those with sleep problems believe it impacts their productivity, which is consistent with a study from Japan by Ishibashi and Shimura [8].

Moreover, the results showed a direct relation between sleep duration and the incidence of sleep problems; people who sleep less than 4 h tend to suffer the most from sleep problems, whereas those who sleep between 7 and 10 h have the best sleep quality. A similar result was found by Hisham et al. and Al-Sayed et al. [20,21]. People tend to use sleeping aids to increase their sleep quality by improving their sleeping hours. 

Our results show that using sleeping aids is high among participants who take more than 60 min to fall asleep compared to those who take between 30 and 60 min. Most people with sleep problems sleep more during the day than at night. People may sleep during the day for various reasons, such as the night shift, studying at night, and late-night activities. This may be related to the increased melatonin secretion at night and its breakdown by sunlight [22]. 

The results of our study showed that a large number of adults use over-the-counter (OTC) sleep pills, perhaps because OTC medications are easy to get and do not require a pre-scription. A study conducted in the USA found that 11.1% of adults used over-the-counter (OTC) sleep medications [23]. This mainly due to people with poor-quality sleep tend to use sleeping aids to help them improve their sleep. Interestingly, we found that most people who consulted a healthcare professional used sleeping aids, unlike those who did not have a consultation. This was consistent with one study that found the number of prescriptions for sleeping pills has increased [24]. 

There are many prescription and nonprescription sleeping aids, such as antihistamines, antidepressants, and benzodiazepines. We found that Panadol Night, which contains an antihistamine, was the most commonly used sleep aid among adults in Madinah. The legal restrictions on other types of sleeping medications and easy access to antihistamines could explain this.

We found that the use of sleeping aids was higher among non-working individuals. This finding aligns with a study conducted in Riyadh that found similar results [20], as people who do not have work might suffer from the difficulty of life and low income. Interestingly, the current results show that the use of sleeping aids among university students is low. In contrast to our results, the use of sleeping aids was high among medical students at King Saud University, Saudi Arabia [21]. This result may be because the study at medical colleges is more challenging than at other colleges.

There are several limitations to our study. Firstly, we did not collect more specific information from participants, such as age and the presence of any underlying medical conditions, which have been shown in previous studies to be important factors associated with sleep problems [24,25]. Secondly, our sample was predominantly female, and previous research has shown that females are more likely to use sleep aids than males [25,26]. This gender imbalance may have influenced our results, and future studies should aim to recruit equal numbers of male and female participants. Thirdly, as a cross-sectional study, our findings are susceptible to recall bias and cannot establish a causal relationship. Lastly, as our study was conducted exclusively in the Madinah region, generalizing our findings to the entire Saudi population may be difficult.

## 4. Materials and Methods

### 4.1. Study Design and Eligibility Criteria

A cross-sectional study of 399 participants was performed in the Madinah region between January 2022 and April 2022, including adults aged 18 years or older who live in the Madinah region and have completed the online questionnaire. However, others outside of the Madinah region and under 18 years old were excluded from the study. 

### 4.2. Sampling Procedure

The Cochrane formula was used to calculate the sample size necessary to achieve a confidence level of 95% with a 5% margin of error and a population of 2.1 million [27]. The estimated required sample size was 385 participants. Cochran’s formula for calculating sample size: *n* = Z2pq/e2. The sampling was conventional, and the questionnaire was disseminated through the WhatsApp messaging app and social media platforms.

### 4.3. Data Collection

Data collection was obtained by using an online questionnaire, which contains three domains. The first domain included general questions regarding demographics such as sex, physical activity, educational level, work status, work hours, nationality, and marital status. The second domain contains questions that evaluate the sleep quality in people, such as sleeping hours, sleeping time, suffering from sleep awakening, sleep onset, napping frequency, causes of trouble sleeping, the effect on productivity and social life, health professional consultation, and sleep aid use. The third domain included only respondents who used a type of sleep aid, which involved questions about the type of sleep aid, the frequency of sleep aid use, the effect of sleep aids on the quality of sleep, the safety of sleep aids, and their recommendation for the possibility of using it.

The degree of clarity and relevance of each item in this questionnaire was systematically validated using the content validity index method (CVI) by sending a questionnaire sample to two experts through email and a hard copy version. According to Davis (1992), the acceptable value is at least 0.80. The questionnaire CVI is 0.91 in relevance and 0.97 in clarity with two experts; therefore, it is validated [28].

### 4.4. Statical Analysis

IBM SPSS 28 for Windows software was used for the analysis, and a *p*-value < 0.05 is considered statistically significant. Descriptive statistics are presented as numbers and percentages for the categorical and continuous variables. Chi-square tests with exact *p*-values were used to compare the characteristics of the participants who use and who do not use sleeping aids. Multiple logistic regression was used to study the association between different factors and using sleeping aids among those having sleep problems. Chi-square and exact tests were used to study the difference between using a sleeping aid and the characteristics of the patients.

### 4.5. Ethical Approval

The survey was carried out with ethical approval from the Institutional Review Board (COPTU-REC-29-20220319) of the vice president of the graduate studies and scientific research department at Taibah University, Madinah region, Saudi Arabia. All participants received a consent form before participating in the study.

## 5. Conclusions

Our study indicates that sleep problems are prevalent in Saudi Arabia. While using sleep aids can improve sleep quality, it is essential to recognize that this is becoming an increasingly significant public health concern in the country. Raising awareness of the importance of sleep quality may enable individuals in Saudi Arabia to better structure their lifestyles and increase productivity. Implementing early detection and intervention programs for people at high risk of experiencing sleep problems is necessary. Further research is required to investigate other regions in Saudi Arabia with a larger and more diverse sample size.

## Figures and Tables

**Table 1:** Characteristics of the participants (N = 399).

		N	%
Sex	Male	125	31.33
	Female	274	68.67
Physical activity	Little or no exercise	267	66.92
	Mild exercise	78	19.55
	Moderate exercise	42	10.53
	Intense exercise	12	3.01
Educational level	High school degree or lower	91	22.81
	Diploma	38	9.52
	Bachelor’s degree	242	60.65
	Masters	26	6.52
	PhD	2	0.50
Work status	Student	96	24.06
	Working	156	39.10
	Not working	147	36.84
How many hours do you work or study?	Less than 4 h	108	27.07
	Between 4 and 7 h	172	43.11
	Between 7 and 10 h	102	25.56
	More than 10 h	17	4.26
Nationality	Saudi	376	94.24
	Non-Saudi	23	5.76
Marital status	Single	122	30.58
	Married	249	62.41
	Divorced	25	6.27
	Widowed	3	0.75
How many hours do you sleep daily (on a typical day)?	Less than 4 h	16	4.01
	4–7 h	271	67.92
	7–10 h	106	26.57
	More than 10 h	6	1.50
On average, what time do you usually go to sleep?	At day	96	24.06
	At night	303	75.94
Do you suffer from continuous sleep awakening?	Yes	239	59.90
	No	160	40.10
How long does it take you to fall asleep once you are in bed?	Less than 30 min	110	27.57
	Between 30 and 60 min	189	47.37
	More than 60 min	100	25.06
Do you take a nap during the day?	No	169	42.36
	Intermittent napping during (week/month) when needed	70	17.54
	Less than 30 min	40	10.03
	30 min to 1.5 h	65	16.29
	2–3 h	46	11.53
	More than 3 h	9	2.26
Do you have trouble sleeping?	Yes	154	38.59
	No	241	61.01

**Table 2:** Characteristics of sleeping problems and using sleeping aid (N = 154).

		N	%
If you have trouble sleeping, what do you think is the cause?	Stress	100	64.94
	Using the phone prior to sleep	57	37.01
	Coffee and tea drinking	51	33.12
	Late activities	30	19.48
	Work	23	14.94
	Studying	23	14.94
	Health condition	17	11.04
	Other	12	7.79
If you have trouble sleeping, did it affect your productivity?	Yes	115	74.68
	No	39	25.32
Have you ever consulted a healthcare professional about your sleep problems?	Yes	23	14.94
	No	131	85.06
Have you used a sleep aid to help you sleep?	Yes	71	46.10
	No	83	53.90

**Table 3:** The association between having sleep troubles and the characteristics of participants.

			Do You Have Trouble Sleeping?		*p*-Value
			Yes (*n* = 154)	No (*n* = 241)	
Sex	Male	N	52	70	0.322
		%	42.6%	57.4%	
	Female	N	102	171	
		%	37.4%	62.6%	
Physical activity	Little or no exercise	N	104	161	0.265
		%	39.2%	60.8%	
	Mild exercise	N	26	52	
		%	33.3%	66.7%	
	Moderate exercise	N	17	24	
		%	41.5%	58.5%	
	Intense exercise	N	7	4	
		%	63.6%	36.4%	
Educational level	High school degree or lower	N	34	56	0.374
		%	37.8%	62.2%	
	Diploma	N	12	26	
		%	31.6%	68.4%	
	Bachelor’s degree	N	95	144	
		%	39.7%	60.3%	
	Master	N	11	15	
		%	42.3%	57.7%	
	PhD	N	2	0	
		%	100.0%	0.0%	
Work status	Student	N	38	54	0.403
		%	41.3%	58.7%	
	Working	N	65	91	
		%	41.7%	58.3%	
	Not working	N	51	96	
		%	34.7%	65.3%	
How many hours do you work or study?	Less than 4 h	N	49	59	0.133
		%	45.4%	54.6%	
	Between 4 and 7 h	N	64	105	
		%	37.9%	62.1%	
	Between 7 and 10 h	N	32	69	
		%	31.7%	68.3%	
	More than 10 h	N	9	8	
		%	52.9%	47.1%	
Nationality	Saudi	N	146	227	0.795
		%	39.1%	60.9%	
	Non-Saudi	N	8	14	
		%	36.4%	63.6%	
Marital status	Single	N	53	65	0.446
		%	44.9%	55.1%	
	Married	N	91	158	
		%	36.5%	63.5%	
	Divorced	N	9	16	
		%	36.0%	64.0%	
	Widowed	N	1	2	
		%	33.3%	66.7%	
How many hours do you sleep daily (on a typical day)?	Less than 4 h	N	13	3	<0.001 *
		%	81.3%	18.8%	
	4–7 h	N	112	157	
		%	41.6%	58.4%	
	7–10 h	N	26	78	
		%	25.0%	75.0%	
	More than 10 h	N	3	3	
		%	50.0%	50.0%	
On average, what time do you usually go to sleep?	In the day	N	53	43	<0.001 *
		%	55.2%	44.8%	
	At night	N	101	198	
		%	33.8%	66.2%	
Do you suffer from continuous sleep awakening?	Yes	N	128	110	<0.001 *
		%	53.8%	46.2%	
	No	N	26	131	
		%	16.6%	83.4%	
Do you take a nap during the day?	No	N	72	95	0.044 *
		%	43.1%	56.9%	
	Intermittent napping during (week/month) when needed	N	20	50	
		%	28.6%	71.4%	
	Less than 30 min	N	16	24	
		%	40.0%	60.0%	
	30 min to 1.5 h	N	18	46	
		%	28.1%	71.9%	
	2–3 h	N	23	22	
		%	51.1%	48.9%	
	More than 3 h	N	5	4	
		%	55.6%	44.4%	
How long does it take you to fall asleep once you are in bed?	Less than 30 min	N	27	81	<0.001 *
		%	25.0%	75.0%	
	Between 30 and 60 min	N	61	126	
		%	32.6%	67.4%	
	More than 60 min	N	66	34	
		%	66.0%	34.0%	

*: Based on the *p*-value, there is a statistical significance between people who have sleep troubles and those who haven’t, in the number of hours they did sleep daily.

**Table 4:** Multiple logistic regression for the factors associated with having sleeping problems.

	OR	*p*-Value	95% C.I. OR	
			Lower	Upper
Sex				
Male	1			
Female	0.74	0.264	0.44	1.25
Work status				
Student	1.00			
Working	1.40	0.420	0.62	3.19
Not working	1.34	0.455	0.62	2.90
Marital status				
Single	1.00			
Married	0.57	0.119	0.29	1.15
Divorced	0.50	0.209	0.17	1.48
Widowed	0.74	0.817	0.06	9.47
Taking a nap during the day				
No	1.00			
Intermittent napping when needed	0.52	0.044	0.28	0.98
Less than 30 min	1.03	0.947	0.49	2.15
30 min to 1.5 h	0.69	0.289	0.35	1.37
2–3 h	1.69	0.149	0.83	3.44
More than 3 h	1.91	0.366	0.47	7.73
Working or studying hours				
Less than 4 h	1.00			
Between 4 and 7 h	0.77	0.406	0.42	1.42
Between 7 and 10 h	0.61	0.168	0.30	1.23
More than 10 h	1.57	0.423	0.52	4.76
Sleeping time				
In day	1.00			
At night	0.41	0.001	0.25	0.69

**Table 5:** Characteristics of using sleeping aids (N = 71).

		N	%
What type of sleep aid have you used?	Panadol night (antihistamine)	35	49.30
	Melatonin	28	39.44
	Herbals/another supplement	17	23.94
	Prescription drugs	8	11.27
	Other	7	9.86
On average, how frequently are you using sleep aids?	As needed	40	56.34
	Daily	20	28.17
	Weekly	7	9.86
	Monthly	1	1.41
	Stop using	3	4.23
Did it help you to improve your sleep quality?	Yes	48	67.6
	No	23	32.4
Do you think it is safe for long-term use?	Yes	20	28.2
	No	51	71.8
Did you notice any side effects?	Yes	27	38.0
	No	44	62.0
Do you recommend using a sleep aid?	Yes	30	42.3
	No	41	57.7

**Table 6:** The association between using sleep aids and characteristics of participants in those having sleeping problems.

			Have You Ever Used a Sleep Aid?		*p*-Value
			Yes N = 71	No N = 83	
Sex	Male	N	24	28	0.993
		%	46.2%	53.8%	
	Female	N	47	55	
		%	46.1%	53.9%	
Physical activity	Little or no exercise	N	48	56	0.664
		%	46.2%	53.8%	
	Mild exercise	N	14	12	
		%	53.8%	46.2%	
	Moderate exercise	N	7	10	
		%	41.2%	58.8%	
	Intense exercise	N	2	5	
		%	28.6%	71.4%	
Educational level	High school degree or lower	N	16	18	0.953
		%	47.1%	52.9%	
	Diploma	N	5	7	
		%	41.7%	58.3%	
	Bachelor’s degree	N	45	50	
		%	47.4%	52.6%	
	Master	N	4	7	
		%	36.4%	63.6%	
	PhD	N	1	1	
		%	50.0%	50.0%	
Work status	Student	N	10	28	0.017
		%	26.3%	73.7%	
	Working	N	33	32	
		%	50.8%	49.2%	
	Not working	N	28	23	
		%	54.9%	45.1%	
How many hours do you work or study?	Less than 4 h	N	28	21	0.294
		%	57.1%	42.9%	
	Between 4 and 7 h	N	27	37	
		%	42.2%	57.8%	
	Between 7 and 10 h	N	13	19	
		%	40.6%	59.4%	
	More than 10 h	N	3	6	
		%	33.3%	66.7%	
Nationality	Saudi	N	68	78	0.726
		%	46.6%	53.4%	
	Non-Saudi	N	3	5	
		%	37.5%	62.5%	
Marital status	Single	N	22	31	0.703
		%	41.5%	58.5%	
	Married	N	45	46	
		%	49.5%	50.5%	
	Divorced	N	4	5	
		%	44.4%	55.6%	
	Widowed	N	0	1	
		%	0.0%	100.0%	
How many hours do you sleep daily (on a typical day)?	Less than 4 h	N	7	6	0.915
		%	53.8%	46.2%	
	4–7 h	N	52	60	
		%	46.4%	53.6%	
	7–10 h	N	11	15	
		%	42.3%	57.7%	
	More than 10 h	N	1	2	
		%	33.3%	66.7%	
On average, what time do you usually go to sleep?	In the day	N	23	30	0.625
		%	43.4%	56.6%	
	At night	N	48	53	
		%	47.5%	52.5%	
Do you suffer from continuous sleep awakening?	Yes	N	59	69	0.996
		%	46.1%	53.9%	
	No	N	12	14	
		%	46.2%	53.8%	
How long does it take you to fall asleep once you are in bed?	Less than 30 min	N	12	15	0.003 *
		%	44.4%	55.6%	
	Between 30 and 60 min	N	19	42	
		%	31.1%	68.9%	
	More than 60 min	N	40	26	
		%	60.6%	39.4%	
Do you take a nap during the day?	No	N	30	42	0.695
		%	41.7%	58.3%	
	Intermittent napping during (week/month) when needed	N	12	8	
		%	60.0%	40.0%	
	Less than 30 min	N	6	10	
		%	37.5%	62.5%	
	30 min to 1.5 h	N	9	9	
		%	50.0%	50.0%	
	2–3 h	N	12	11	
		%	52.2%	47.8%	
	More than 3 h	N	2	3	
		%	40.0%	60.0%	
If you have trouble sleeping, did it affect your productivity?	Yes	N	56	59	0.353
		%	48.7%	51.3%	
	No	N	15	24	
		%	38.5%	61.5%	
Have you ever consulted a healthcare professional about your sleep problems?	Yes	N	20	3	<0.001 *
		%	87.0%	13.0%	
	No	N	51	80	
		%	38.9%	61.1%	

*: Based on the *p*-value, there is a statistical significance between people who used sleep aids and who did not, in the required time to fall asleep once they are in their beds.

**Table 7:** Multiple logistic regression for the factors associated with using sleep aids among those having sleeping problems.

	OR	*p*-Value	95% CI for OR	
Work status				
Student	1.00			
Working	2.88	0.032	1.10	7.55
Not working	4.31	0.005	1.56	11.91
How long does it take you to fall asleep once you are in bed?				
More than 60 min	1.00			
Less than 30 min	0.40	0.075	0.14	1.10
Between 30 and 60 min	0.27	0.002	0.12	0.61
Have you ever consulted a healthcare professional about your sleep problems?				
No	1.00			
Yes	10.96	<0.001	2.87	41.87

## Data Availability

Data are available by contacting the corresponding author upon reasonable request.
